# Storage Stability of Blood Samples for miRNAs in Glycosylated Extracellular Vesicles

**DOI:** 10.3390/molecules29010103

**Published:** 2023-12-23

**Authors:** Cuidie Ma, Rui Ding, Kun Hao, Wenqian Du, Lida Xu, Qi Gao, Changyuan Yu

**Affiliations:** 1 College of Life Science and Technology, Beijing University of Chemical Technology, Beijing 100029, China; 18301510262@163.com; 2 Department of Laboratory Medicine, State Key Laboratory of Complex Severe and Rare Diseases, Peking Union Medical College Hospital, Chinese Academy of Medical Science and Peking Union Medical College, Beijing 100730, China; drdingrui@163.com; 3 Beijing Hotgen Biotech Co., Ltd., Beijing 102600, China; kun.hao@hotgen.com.cn (K.H.); wenqian.du@hotgen.com.cn (W.D.); lida.xu@hotgen.com.cn (L.X.)

**Keywords:** glycosylated extracellular vesicles, microRNAs, stability, blood

## Abstract

Extracellular vesicle (EV) miRNAs are promising biomarkers for clinical diagnosis. However, their stability is a crucial concern affecting reliability and accuracy. Factors such as sample collection, processing, storage conditions, and experimental procedures impact EV miRNA stability. Studying EV miRNA stability aims to find optimal handling and storage methods, ensuring integrity and functionality throughout research. In this study, we used RT-qPCR and GlyExo-Capture technology, which can specifically capture glycosylated EVs by lectin, to assess the stability of glycosylated EV miRNAs. We found that slow acceleration centrifugation and two-step centrifugation methods were suitable for subsequent experiments. To ensure uniformity, we recommend using the two-step centrifugation method. We also studied blood storage before serum separation and recommend separation within 2 h at 4 °C or 25 °C. For separated serum samples, higher temperatures accelerated miRNA degradation, and the storage duration should be adjusted based on laboratory conditions. Short-term storage at −20 °C is acceptable for up to 3 months while avoiding repeated freeze–thaw cycles. We developed protective agents to extend the storage time at 25 °C, meeting clinical requirements. Additionally, Lakebio’s cfRNA storage tubes effectively preserved the stability of miRNAs in plasma glycosylated EVs. Understanding EV miRNA stability provides insights into optimizing sample handling, storage strategies, and enhancing reliability in clinical applications.

## 1. Introduction

EVs are diverse lipid-based membrane-bound vesicles of varying sizes and contents released by different cells [1]. Extensive research has demonstrated that EVs can be derived from various body fluids, including blood [2], urine [3], cerebrospinal fluid [4], breast milk [5], malignant ascites [6], saliva [7], tears [8], semen [9], and amniotic fluid [10]. The cargo of EVs encompasses a wide range of molecules, such as lipids, proteins, metabolites, DNA, mRNA, miRNA, and other non-coding RNAs [1]. EVs efficiently transfer their cargo from donor cells to recipient cells, facilitating intercellular communication and delivering genetic material and signaling moieties through multiple pathways. This process plays a crucial role in regulating diverse physiological processes, including tissue repair, tumor diagnosis and treatment, and immune modulation [11,12].

Aberrant glycosylation, characterized by abnormal modifications of glycoproteins, plays a major role in tumor cell growth, invasion, metastasis, and immune evasion [13]. Notably, studies have revealed that tumor-derived EVs are abundantly covered with glycoproteins, which can also exhibit aberrant glycosylation patterns. The isolation and analysis of aberrantly glycosylated EVs hold great promise for the discovery of novel biomarkers applicable to tumor diagnosis [14]. The presence of aberrant glycosylation in EVs has been associated with disease progression, underscoring its significant potential as a diagnostic biomarker [15].

miRNAs are a class of small RNAs, typically consisting of approximately 20–24 nucleotides in length. These miRNAs exert regulatory effects by binding to target mRNAs, leading to the modulation of gene expression through translational inhibition or mRNA degradation. Primarily found in eukaryotes, miRNAs are integral to the intricate regulation of numerous biological processes, including cell differentiation, proliferation, apoptosis, and other events closely intertwined with disease occurrence and development [16]. Recent research has shed light on the fact that cells can release miRNAs via EVs, thereby influencing the growth of neighboring cells. miRNAs in EVs play major roles in various biological processes, such as intercellular communication, disease diagnosis and treatment, tumor development, and immune regulation [17].

miR-125a is a miRNA residing within EVs that exerts regulatory control over the gene expression of target cells. Multiple studies have indicated that miR-125a in EVs can impede the growth and metastasis of tumor cells by targeting specific genes, including SMAD2, SMAD4, and TRAF6 [18]. In a similar vein, miR-150 in EVs assumes a crucial role in influencing immune system regulation and the immune response by modulating the function of immune cells. Research has demonstrated that miR-150 in EVs can be effectively transferred to target cells, thereby impacting the gene expression and function of these cells. Consequently, miR-150 influences the proliferation and differentiation of T cells, thereby orchestrating immune processes [19]. For normalization of miRNA expression levels in EVs, miR-16 and let-7a are commonly utilized as internal reference genes. These genes are renowned for their minimal expression changes across different conditions and considered as stable reference standards [20,21].

EVs have emerged as a compelling avenue for diagnostics due to their abundant presence in various body fluids. Unlike invasive surgical biopsies that provide limited information from a specific tissue site, EVs offer a non-invasive and continuous sampling approach, enabling the acquisition of systemic information with broad diagnostic potential. However, the inherent challenge lies in the fact that blood, a commonly utilized source of EVs, is highly susceptible to rapid deterioration in vitro [22,23]. While miRNAs encapsulated within EVs generally exhibit greater stability compared with free miRNAs, they are not entirely impervious to degradation under specific conditions. EVs harbor nucleases that possess the ability to catalyze the degradation of miRNAs. Moreover, the intricate fluidic environment of EVs introduces additional factors such as RNases, proteases, and other molecules that can bind to or directly catalyze the degradation of miRNAs, thus influencing their stability [24].

The effective preservation of EV contents is pivotal to ensure the quality and diagnostic value of acquired biomarkers [25]. While fresh samples are universally preferred [26], the immediate separation of EVs from collected patient blood samples is often unfeasible. All types of blood cells continuously release EVs and miRNAs into the extracellular environment. Inadequate blood handling may lead to cell contamination or hemolysis of plasma or serum, inducing alterations in miRNA expression profiles unrelated to disease [27]. Extensive research has unveiled the impact of peripheral blood processing, sample storage, and variations in processing steps on EV yield, composition, and functionality. Factors such as the choice of anticoagulant, sample collection or processing time, and separation methods have been demonstrated to significantly influence EV yield and blood biomarkers [26,28,29]. Therefore, blood handling and storage become crucial.

Before isolating EV miRNAs, careful consideration of sample storage and processing methods is imperative. The feasibility of utilizing samples stored long term for research purposes, given the presence of RNase activity, remains uncertain. In this study, we employed the GlyExo-Capture technology, previously developed for the specific capture of fucosylated EVs, in conjunction with RT-qPCR to determine the miRNAs in fucosylated Evs. Our aim was to explore the optimal sample handling steps, sample storage conditions, and storage time of samples during the isolation of glycosylated EV miRNAs in clinical experiments, ultimately ensuring the stability of glycosylated EV miRNAs.

## 2. Results

### 2.1. Glycosylated EV Enrichment Based on the GlyExo-Capture Method

**Figure 1:**
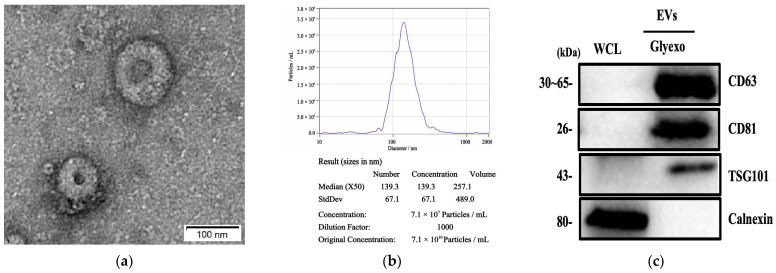
Characterization of serum glycosylated EVs. (**a**) Transmission electron microscopy (TEM) image of glycosylated EVs (bar = 100 nm). (**b**) Nanoparticle tracking analysis (NTA) of glycosylated EVs. (**c**) Western blot analysis of unenriched and exosomesenriched proteins in glycosylated EVs and whole cell lysate (WCL).

Previous studies have indicated that the GlyExo-Capture technique can effectively capture glycosylated EVs. In this study, to investigate the stability of serum glycosylated EVs, we initially characterized the isolated glycosylated EVs from healthy donors through transmission electron microscopy (TEM), nanoparticle tracking analysis (NTA), and Western blot (WB). Results revealed that the isolated glycosylated EVs had an average diameter of 100 nm, displaying distinct double-layered membrane and cup-shaped structures. NTA measurements demonstrated a size distribution of 153.1 ± 67.1 nm, consistent with the expected size range of EVs (Figure 1a,b). Western blot analysis confirmed the presence of the EV marker proteins CD63, CD81, and TSG101, while the negative control marker calnexin was not detected (Figure 1c). These comprehensive findings provide evidence for the successful isolation of EVs from serum samples using GlyExo-Capture technology.

### 2.2. Comparison of Glycosylated EV miRNAs in Different Serum Separation Methods

**Figure 2:**
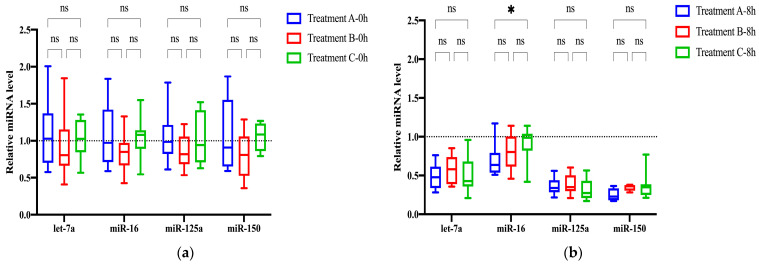
Selection of serum separation methods. (**a**) Relative expression levels of four glycosylated EV miRNAs in three different serum separation methods at 0 h. (**b**) Relative expression levels of four glycosylated EV miRNAs in three different serum separation methods at 8 h. Treatment A: two-step centrifugation, Treatment B: rapid acceleration centrifugation, Treatment C: slow acceleration centrifugation. * *p* < 0.05, ns: nonsignificant.

To minimize the presence of cellular debris, we evaluated the effects of three different centrifugation processes on serum glycosylated EV miRNAs. Treatment A underwent two-step centrifugation, Treatment B underwent rapid acceleration centrifugation, and Treatment C underwent slow acceleration centrifugation. Among the three fresh serum isolation processes, the difference in the relative expression levels of the four glycosylated EV miRNAs, including miR-16, let-7a, miR-125a, and miR-150, was not significant (Figure 2a). Detailed statistical analysis information is provided in Table S7.

Considering the demanding workload in clinical hospitals, where serum samples are not immediately processed and aliquoted after centrifugation, we investigated the degradation of serum samples left at 25 °C for 8 h after centrifugation. The degradation levels of the four glycosylated EV miRNAs ranged from 5% to 77% across the three treatments. Specifically, Treatment A exhibited degradation ranging from 36% to 77%, Treatment B displayed degradation ranging from 5% to 58%, and Treatment C showed degradation ranging from 13% to 70% (Figure 2b, Table S2). The extent of miRNA degradation varied among different miRNAs, with low-abundance miRNAs being more susceptible to degradation compared with high-abundance miRNAs. Treatment B demonstrated less degradation compared with Treatment A and Treatment C, possibly due to the initially lower relative expression levels of miRNAs in Treatment B. Overall, there were no significant differences in the relative expression levels of miRNAs between the slow-speed centrifugation and the two-step centrifugation methods at either 0 h or 8 h. Both processes are suitable for serum EV isolation. However, considering the use of diverse centrifuge models in clinical settings, some of which may not support slow acceleration, the two-step centrifugation method was chosen for subsequent experiments in this study.

### 2.3. Impact of Storage Conditions on Glycosylated EV miRNAs before Serum Separation

**Figure 3:**
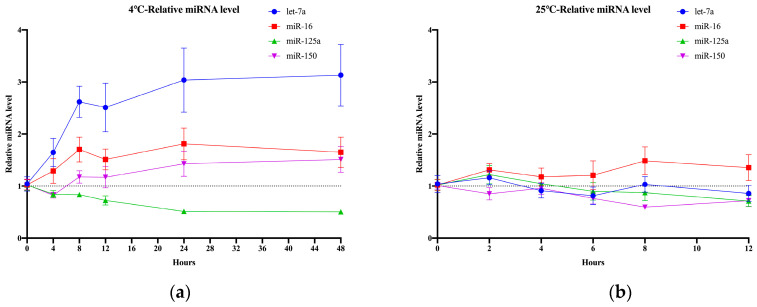
Storage conditions before serum separation. (**a**) Relative expression levels of four glycosylated EV miRNAs under different storage times before serum separation at 4 °C. (**b**) Relative expression levels of four glycosylated EV miRNAs under different storage times before serum separation at 25 °C.

In light of the demanding workload in clinical hospitals, we conducted experiments to evaluate the degradation of blood samples under varying storage durations before serum separation at both 4 °C and 25 °C. Hemolysis occurred in the collection tubes at 4 °C after 4 h. Although it was observed in Figure 3a that the relative expression levels of miR-16, let-7a, and miR-150 increased, and those of miR-125a decreased, there was no significant difference (Figure 3a). Detailed statistical analysis information is provided in Table S8. At 25 °C, the relative expression levels of the four miRNAs remained stable for up to 12 h (Figure 3b). Previous experiments have shown that isolated serum experienced degradation ranging from 36% to 77% after 8 h at 25 °C, while miRNAs in unseparated whole blood remained stable. This observation reflects the dynamic balance between the continuous secretion of EVs by cells and the degradation of miRNAs. Consequently, it is crucial to promptly separate the serum from blood samples, ideally within 2 h under both 4 °C and 25 °C conditions.

### 2.4. Impact of Serum Storage Conditions on Glycosylated EV miRNAs

**Figure 4:**
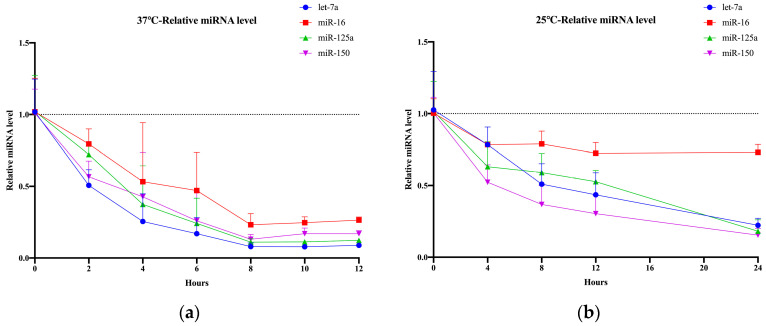

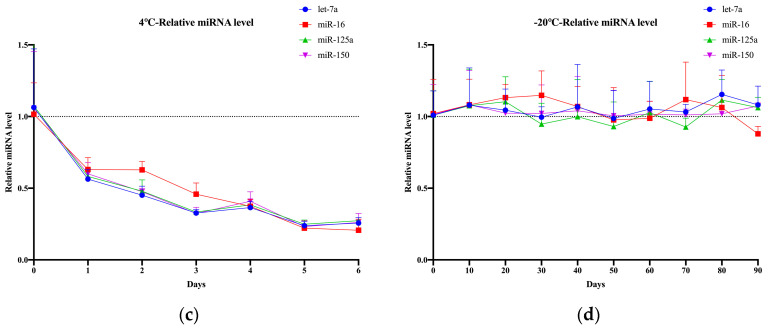
Storage conditions of serum. (**a**) Relative expression levels of four glycosylated EV miRNAs under different storage times after serum separation at 37 °C. (**b**) Relative expression levels of four glycosylated EV miRNAs under different storage times after serum separation at 25 °C. (**c**) Relative expression levels of four glycosylated EV miRNAs under different storage times after serum separation at 4 °C. (**d**) Relative expression levels of four glycosylated EV miRNAs under different storage times after serum separation at −20 °C.

Due to space limitations and the limited availability of −80 °C freezers, many hospitals and laboratories store serum samples at −20 °C or even 4 °C for extended periods. We evaluated the storage duration of separated serum samples at temperatures of 25 °C, 37 °C, 4 °C, and −20 °C. Detailed statistical analysis information is provided in Table S9. After 8 h at 25 °C, the four glycosylated EV miRNAs experienced varying degrees of degradation, ranging from 21% to 63%, with miR-16 degrading by 27% after 24 h, and the other three miRNAs degrading by approximately 80% (Figure 4a). The extent of degradation varied among the different miRNAs, with low-abundance miRNAs exhibiting a higher degree of degradation compared with high-abundance miRNAs. At 37 °C, the four glycosylated EV miRNAs degraded by 22% to 50% after 2 h, and the degradation ranged from 48% to 75% after 4 h, similar to the degradation observed after 24 h at 25 °C (Figure 4b). At 4 °C, miR-16 degraded by 38% after 1–2 days, let-7a degraded by approximately 50%, and, after 6 days, miR-16 degraded by 80% while let-7a degraded by 76% (Figure 4c). Detailed results are provided in Tables S3–S5. Serum samples stored at −20 °C maintained stable relative expression levels of the four glycosylated EV miRNAs for up to 90 days (Figure 4d). Therefore, serum intended for glycosylated EV miRNA extraction can be stored at −20 °C for a period of 3 months. Higher temperatures result in faster degradation of glycosylated EV miRNAs. Considering the weather and seasonal factors in clinical experiments, shorter timeframes for serum separation are required during the summer compared with winter. Short-term storage can be performed at −20 °C for up to 3 months.

### 2.5. Effect of Freeze–Thaw Cycles on Glycosylated EV miRNAs in Serum

**Figure 5:**
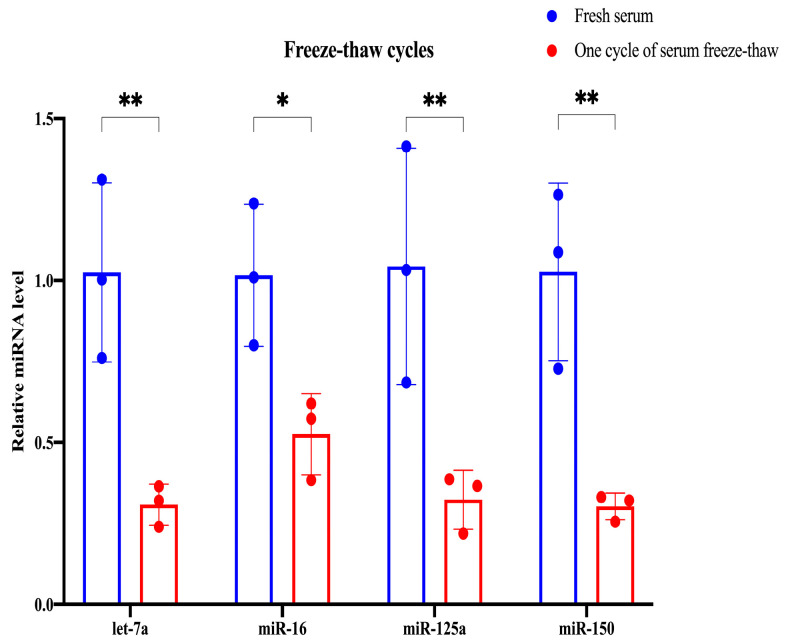
The impact of freeze–thaw cycles on the relative expression levels of four glycosylated EV miRNAs. * *p* < 0.05, ** *p* < 0.01.

The stability of glycosylated EV miRNAs in serum is significantly affected by freeze–thaw cycles. We assessed the relative expression levels of glycosylated EV miRNAs in fresh serum and serum subjected to a single freeze–thaw cycle. Following one freeze–thaw cycle, miR-16 degraded by 48% and let-7a, miR-125a, and miR-150 degraded by 70% compared with fresh serum (Figure 5). Detailed statistical analysis information is provided in Table S10. Consequently, it is imperative to avoid repeated freeze–thaw cycles for serum intended for glycosylated EV miRNA studies, with storage limited to a single freeze–thaw cycle at −80 °C.

### 2.6. Protection of Glycosylated EV miRNAs in Serum by Adding Protectants

**Figure 6:**
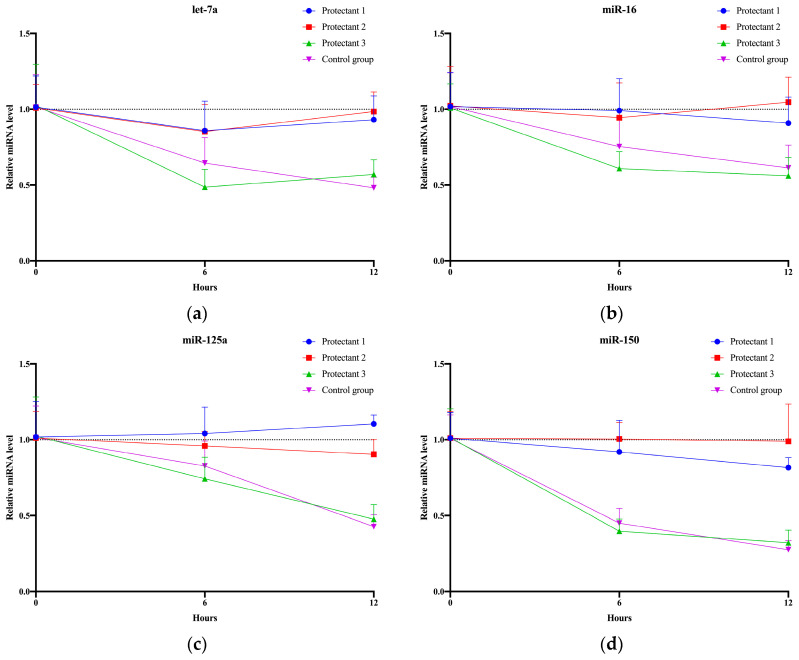
The impact of protectants on serum storage conditions. (**a**) Relative expression levels of let-7a in serum glycosylated EVs under different storage times using three groups of protectants at 25 °C. (**b**) Relative expression levels of miR-16 in serum glycosylated EVs under different storage times using three groups of protectants at 25 °C. (**c**) Relative expression levels of miR-125a in serum glycosylated EVs under different storage times using three groups of protectants at 25 °C. (**d**) Relative expression levels of miR-150 in serum glycosylated EVs under different storage times using three groups of protectants at 25 °C.

Due to the demanding nature of clinical hospital work, large sample volumes, and remote transportation, separated serum often needs to be stored at room temperature for a certain duration. Our previous investigations revealed that serum undergoes degradation to some extent at both 25 °C and 37 °C. Hence, we developed a formulation for a protective agent to be added to separated serum and evaluated its impact on glycosylated EV miRNAs (Figure 6a–d). Detailed statistical analysis information is provided in Table S11. After 12 h at 25 °C, the control group exhibited varying degrees of degradation ranging from 40% to 73% across the four tested miRNAs. Among the protectant groups, Protectant 1, containing a 200 mM ribonucleoside–vanadyl complex (RVC) without PC300, showed the highest degradation for miR-150 (19%). Protectant 2, containing a 200 mM RVC with PC300, exhibited the highest degradation for miR-125a (11%). Protectant 3, which involved the addition of normal saline, demonstrated degradation ranging from 44% to 68% across the four tested miRNAs. Detailed results can be found in Table S6. These experiments demonstrated that the formulated protective agents can prolong the storage time of serum at room temperature, thereby meeting the transportation and operational requirements of clinical experiments.

### 2.7. Comparison of Glycosylated EV miRNAs in Plasma Separated by Three Different Collection Vessels

**Figure 7:**
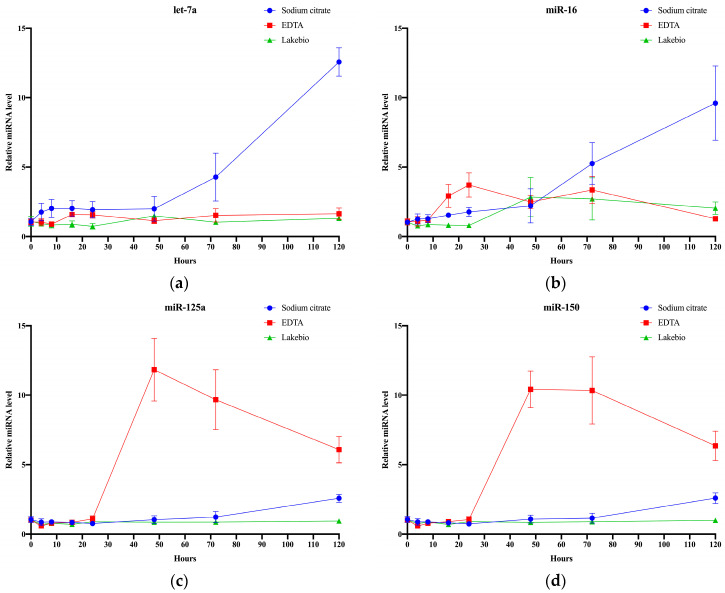
Comparison of three different plasma collection vessels. (**a**) Relative expression levels of let-7a in glycosylated EVs under different storage times using three different plasma collection vessels. (**b**) Relative expression levels of miR-16 in glycosylated EVs under different storage times using three different plasma collection vessels. (**c**) Relative expression levels of miR-125a in glycosylated EVs under different storage times using three different plasma collection vessels. (**d**) Relative expression levels of miR-150 in glycosylated EVs under different storage using three different plasma collection vessels.

To evaluate the protective effects of various components on glycosylated EV miRNAs in plasma, we collected fresh blood from healthy individuals using three different plasma collection tubes (Figure 7a–d). Detailed statistical analysis information is provided in Table S12. The relative expression level of miR-16 in EDTA plasma collection tubes showed a significant increase after 8 h, followed by a subsequent decline. There were no significant differences in the expression of let-7a throughout the experimental time. The relative expression levels of miR-125a and miR-150 significantly increased after 48 h. In sodium citrate plasma collection tubes, the relative expression levels of miRNAs remained stable at the beginning of the experiment, with a significant increase observed for let-7a and miR-16 after 72 h that continued until the end of the experiment. The relative expression levels of miR-125a and miR-150 showed an increasing trend after 72 h, but without significant differences. Lakebio’s cfRNA preservation tubes maintained stable relative expression levels of miRNAs for up to 120 h. Therefore, in this study, the utilization of Lakebio’s cfRNA preservation tubes proved to be superior in preserving the integrity and stability of glycosylated EV miRNAs in plasma.

## 3. Discussion

EVs are distributed in diverse bodily fluids, rendering them more accessible compared with tumor cells. The characteristics of EVs are influenced by their cellular origins and their involvement spans various facets of the body’s immune response, antigen presentation, cell migration, cell differentiation, and tumor invasion. Consequently, if EVs and their cargo demonstrate notable in vitro stability, they could emerge as the most promising and readily available biomarkers for cancer gene detection. EVs secreted by tumor cells display distinct types and quantities in comparison with those derived from normal cells, with tumor-secreted EVs being significantly more abundant. Consequently, tumor-specific EVs within bodily fluids hold great potential as highly promising diagnostic markers for cancer [30]. Aberrant glycosylation represents a pivotal characteristic of tumor cell malignancy, reflecting abnormalities in cellular epigenetics and gene expression associated with polysaccharide biosynthesis [31]. Studies have demonstrated that certain types of polysaccharides exhibit preferential enrichment on EVs relative to the diverse range of polysaccharide types found in cell membranes [15]. We employed the GlyExo-Capture technique to capture glycosylated EVs by affinity separation of specific sugar chains using phytolectins. This approach was employed for the selection of tumor-related targets, providing experimental evidence for early tumor diagnosis, disease monitoring, and prognosis. In the present study, we assessed the impact of blood collection, processing, and storage conditions on glycosylated EV miRNAs. This encompassed the examination of serum separation methods, pre- and post-separation storage conditions, freeze–thaw cycles, and the utilization of serum protectants. Additionally, we investigated the influence of different plasma collection tubes on the stability of glycosylated EV miRNAs within the plasma milieu.

Among the three serum separation methods, the rapid acceleration centrifugation group exhibited a minimal amount of residual red substance on the gel layer, which may have an impact on the determination of EVs in the serum. Conversely, the slow acceleration centrifugation group and the two-step centrifugation group did not show any residual red phenomena. Studies have indicated that platelet activation in serum may also lead to a significant increase in EVs. Different miRNAs are influenced to varying degrees by hemolysis and platelet activation [32,33]. Hence, we hypothesize that the visible red residue in the rapid acceleration centrifugation group may be attributed to the presence of broken red blood cells, leading to the generation of cellular debris or content release. Consequently, after a period of room temperature incubation, the content of EVs increases, offsetting the degradation and yielding a result indicative of a lower degradation rate. To minimize the presence of cell debris and reduce cell contamination, we recommend employing the two-step centrifugation method for experiments.

**Figure 8:**
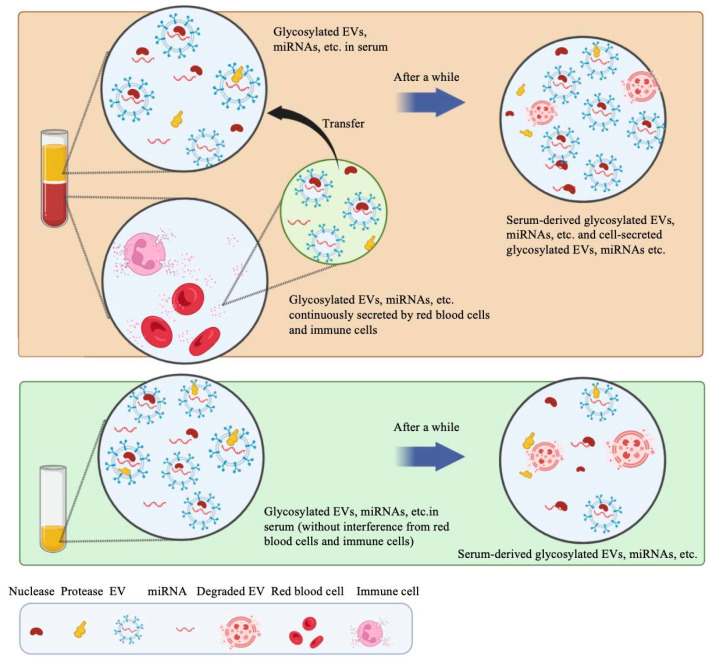
Diagram of intercellular communication of glycosylated EV miRNAs at 25 °C. After a certain period, glycosylated EVs and nucleic acids in serum undergo degradation. However, cells continuously secrete glycosylated EVs or release apoptotic bodies and nucleic acids through apoptosis, maintaining a balance of nucleic acid levels in the serum. When serum is separated from blood cells and transferred to a new tube, glycosylated EVs degrade in the serum after a while, leading to a reduction in nucleic acid levels.

In our analysis, the immediate separation of serum after blood collection was of utmost importance, aligning with the requirements for other clinical indicators [26,34,35]. Under the condition of 25 °C, the relative expression [36] levels of glycosylated EV miRNAs remained stable in the collection tubes. This stability can be attributed to the communication between blood cells and serum. After a certain period, glycosylated EVs and nucleic acids in serum undergo degradation. However, cells continuously secrete glycosylated EVs or release apoptotic bodies and nucleic acids through apoptosis, maintaining a balance of nucleic acid levels in the serum. When serum is separated from blood cells and transferred to a new tube, glycosylated EVs degrade in the serum after a while, leading to a reduction in nucleic acid levels. Without the secretion of substances such as cell-secreted glycosylated EVs, nucleic acids exhibit a declining trend. This phenomenon is depicted in Figure 8. However, storing samples at 4 °C for 4 h resulted in hemolysis, disrupting the equilibrium state of miRNAs. Recent studies have identified 287 known and 72 putative novel miRNAs in red blood cells. Notably, miR-451a, miR-144-3p, miR-16, miR-92a, let-7, and miR-486-5p exhibited the highest expression levels in red blood cells [37]. Moreover, research has linked miR-150 to storage lesions in red blood cells [38,39]. While miR-125 exhibits relatively low expression levels in red blood cells, its specific mechanism of action within these cells remains incompletely understood. Consequently, it is plausible that the relative expression levels of let-7a, miR-16, and miR-150 are increased, while the relative expression level of miR-125 is decreased.

For long-term storage of EVs spanning several days to months, it is widely accepted that freezing at −80 °C or below is the recommended approach [34]. Accordingly, in this experiment, samples underwent various treatments before being stored at −80 °C. Several studies have indicated that the DNA content and functionality of EVs remain stable under conditions of 4 °C, room temperature, and repeated freeze–thaw cycles [40]. However, investigations on the stability of glycosylated EV miRNAs at different temperatures have produced conflicting results. Some studies have reported the relative stability of glycosylated EV miRNAs [41,42], while a study by Toshiko Aiso reported contradictory findings [43]. The latter study is noteworthy due to its striking similarity to the findings reported here, specifically regarding the instability of miRNAs and their varying rates of degradation. Differences between these studies can be attributed to the limited number of tested miRNAs, the utilization of human serum/plasma, or relatively short analysis time.

Most studies indicate that multiple freeze–thaw cycles of samples can affect the characteristics and concentration of EVs [26,44,45]. Platelet activation and fragmentation contribute to the production of EVs [46,47,48]. Research suggests that, in plasma samples subjected to a single centrifugation, freezing and thawing can lead to an increase in EV content, whereas in platelet-depleted plasma samples obtained after two-step centrifugation there is no significant difference in EV content before and after freezing [33]. However, in our study, freeze–thaw cycles resulted in a significant decrease in EV miRNAs, possibly due to different detection methods. Unlike the vesicles themselves, our study focused on their contents. Freeze–thaw cycles do not show a positive correlation with the content and size of EVs. Multiple freeze–thaw cycles may lead to membrane damage and fusion in EVs, along with content leakage, as reported in the literature [49,50]. While safeguarding the integrity of EV miRNAs, we aim to minimize the ongoing release of non-disease-related EVs from other blood cells, mitigate the adverse effects of freeze–thaw cycles, and strive to avoid repeated freeze–thaw cycles [51].

After observing alterations in stored samples, we attempted to mitigate the impact of storage on EV miRNAs by introducing a protective agent. Some authors have focused their research on low-temperature protective agents such as DMSO and trehalose. However, the protective effects vary among these agents [52,53,54]. In this study, our emphasis was on preserving EV miRNAs. We introduced RVC as a protective agent. RVC is a transition metal compound known to inhibit various RNase activities and is suitable for maintaining the integrity of RNA molecules in the buffer solution. The addition of RVC significantly slowed down the glycosylation-induced degradation of EV miRNAs, providing more time for the processing and transportation of serum. In conclusion, we demonstrated that glycosylated EV miRNAs can remain stable under the protection of RVC.

While our focus has been on serum samples, many studies analyze EVs in plasma. After testing serum preservatives, we sought to identify suitable blood collection tubes in plasma to prolong the stability of glycosylated EV miRNAs. As blood cells and plasma were not separated, a dynamic equilibrium was reached in the early stages of the experiment, with cells continuously secreting EVs [55], while those in the plasma underwent degradation. In comparison with sodium citrate plasma collection tubes, EDTA plasma collection tubes disrupted the balance earlier, leading to an initial dominance of cell-secreted EVs. Subsequently, the balance shifted, and the relative expression of miRNAs started to decline as the secretion of EVs from cells became less than the degradation of EVs. Sodium citrate plasma collection tubes disrupted the balance later, with cell-secreted EVs outnumbering degraded EVs during the experiment, resulting in an increasing trend. This difference may be attributed to the binding of EDTA with calcium ions, affecting cellular calcium ion channels and, consequently, EV release [56,57].

Previous research indicated that storing blood in EDTA collection tubes for 6 h maintained stable EV concentrations, while sodium citrate plasma collection tubes increased EV concentrations by 1.5-fold [58]. According to the data in Supplementary Table S12, in our study, after storing blood for 8 h in sodium citrate plasma collection tubes and EDTA plasma collection tubes, the average relative expression levels of EV miRNAs were 1.24-fold and 0.91-fold, respectively. This aligns with the previous study, but our experiment was extended over a longer duration. After 72 h, neither collection tube prevented the release of platelet-activated and platelet-derived EVs. Lakebio tubes utilize EDTA as an anticoagulant and incorporate metabolic inhibitors, such as sodium azide, sodium fluoride, and glyoxal, to suppress cellular metabolism, inhibit the mitochondrial respiratory chain, block apoptotic and necrotic pathways, and protect EVs in blood. This preservation method ensures the integrity and stability of miRNAs, effectively suppressing nucleolytic activity in blood samples and preventing the non-targeted release of background blood cell genomic RNA. It also maintains the integrity and stability of cell morphology, contributing to reliable sample quality. For detailed product specifications, please refer to the webpage http://lakebio.com/ylrnacwbcg, accessed on 1 July 2023.

This study focused on the analysis of four specific miRNAs, including miR-16 and let-7a, which are widely recognized as internal references, and miR-125a and miR-150, which are associated with tumor-related pathways. While the selected set encompasses both high- and low-abundance miRNAs, it is crucial to acknowledge the limited number of miRNAs investigated. Consequently, the findings may not fully represent the comprehensive expression profile of all miRNAs. Future research endeavors with a more extensive selection of miRNAs are warranted to provide a more holistic understanding of miRNA expression patterns.

In conclusion, we have comprehensively assessed the influence of blood collection, processing, and storage conditions on glycosylated EV miRNAs. It is of utmost importance to meticulously maintain the integrity and dependability of samples throughout the entire workflow, encompassing collection, transportation, and storage stages. These meticulous measures are indispensable for guaranteeing the accuracy and reproducibility of experimental outcomes. Furthermore, the preservation of stability serves as a protective measure for the integrity of biomarkers, effectively averting their degradation and inactivation and ensuring the dependable reflection of pertinent biological processes or disease statuses within clinical research. The findings of this study provide guidance for ensuring the reliability of glycosylated EV miRNAs extracted from blood samples in clinical practice. Additionally, a comprehensive understanding of the impact of collection, processing, and storage conditions on these biomolecules contributes to enhancing their accuracy and reliability in disease diagnosis. Ensuring the accuracy of experimental results during the drug development phase is also of paramount importance in the development of therapeutic approaches based on blood-sample-derived EV miRNAs.

## 4. Materials and Methods

### 4.1. Blood Collection

This study was conducted in accordance with the Declaration of Helsinki and approved by the Ethics Committee of Peking Union Medical College Hospital (JS-3449D). This study involved the collection of blood samples from a cohort of healthy volunteers, and specific inclusion and exclusion criteria were applied to ensure the homogeneity of the study population. Inclusion criteria mandated a minimum age of 18 years, while exclusion criteria encompassed conditions such as pregnancy, immunosuppression, other cancers, or initiation of palliative treatment.

### 4.2. Serum/Plasma Separation

#### 4.2.1. Comparison of Glycosylated EV miRNAs in Different Serum Separation Methods

Fresh blood samples were aseptically collected from a cohort of 10 healthy individuals using disposable serum collection tubes (BD, Franklin Lakes, NJ, USA). Each individual provided three copies and divided the copies into three different treatments. All sample processing procedures were completed within a minimum of 30 min to a maximum of 90 min to maintain sample integrity.

Treatment A (two-step centrifugation): The blood sample was centrifuged at the end of the clotting time (30–90 min) in a horizontal rotor (swing-out head) for 10 min at 1800× *g* at room temperature. Following centrifugation, 600 μL of the serum was kept in the original serum collection tube, and the remaining serum was collected in a separate 2 mL centrifuge tube. The collected serum underwent a second centrifugation for 10 min at 3000× *g* at room temperature. The resulting supernatant was transferred into 1.5 mL centrifuge tubes (550 μL per tube). The remaining serum in the original tube was preserved at 25 °C for 8 h, followed by a second centrifugation for 10 min at 3000× *g* at room temperature. Finally, the serum was transferred to a 1.5 mL centrifuge tube for further analysis.

Treatment B (rapid acceleration centrifugation): Another copy of the blood sample was centrifuged in a horizontal rotor (swing-out head) for 10 min at 3000× *g* with rapid acceleration (mode 9) at room temperature. Similarly, 600 μL of the serum was kept in the original serum collection tube, and the remaining serum was collected in a 1.5 mL centrifuge tube (550 μL per tube). The serum in the original tube was preserved at 25 °C for 8 h before being transferred to a 1.5 mL centrifuge tube.

Treatment C (slow acceleration centrifugation): The third copy of the blood sample was centrifuged in a horizontal rotor (swing-out head) for 10 min at 3000× *g* in the slow acceleration mode at room temperature. After centrifugation, 600 μL of the serum was kept in the original serum collection tube, and the remaining serum was collected in a 1.5 mL centrifuge tube (550 μL per tube). The serum in the original tube was preserved at 25 °C for 8 h and then transferred into a 1.5 mL centrifuge tube. Subsequently, the separated serum samples were promptly stored at −80 °C for long-term stability during subsequent experimental analyses.

#### 4.2.2. Impact of Storage Conditions on Glycosylated EV miRNAs before Serum Separation

Fresh blood samples were aseptically obtained from a cohort of 10 healthy individuals using disposable serum collection tubes (BD, NJ, USA). Each individual contributed a total of six tubes of blood, with 3 mL to 5 mL of blood in each tube. The blood of five individuals was placed at 4 °C for 0, 4, 8, 12, 24, and 48 h, and the blood of the other five individuals was stored at 25 °C for 0, 4, 6, 8, 10, and 12 h. Following the designated time intervals, the blood collection tubes were subjected to two-step centrifugation to facilitate the separation of the serum. The resulting serum was carefully collected and stored at −80 °C, ensuring optimal preservation for subsequent experimental investigations.

#### 4.2.3. Impact of Serum Storage Conditions on Glycosylated EV miRNAs

Fresh blood samples were collected from a cohort of 12 healthy individuals using disposable serum collection tubes (BD, Franklin Lakes, NJ, USA). Each individual provided two tubes of blood and mixed two tubes into 7 mL to 10 mL of blood. The serum separation process, employing the two-step centrifugation method, was carried out promptly within a minimum of 30 min to a maximum of 90 min to minimize any potential alterations in sample integrity. Following serum separation, the samples were subjected to various storage conditions to assess the effects of different temperatures and durations on sample stability. The 12 healthy individuals were evenly divided into 4 groups for the following experiments, with 3 biological replicates for each group. Specifically, the serum samples were stored at the following conditions: 25 °C for 0, 4, 8, 12, 24, and 48 h; 37 °C for 0, 2, 4, 6, 8, 10, and 12 h; 4 °C for 0, 1, 2, 3, 4, 5, and 6 days; and −20 °C for 0, 10, 20, 30, 40, 50, 60, 70, 80, and 90 days. At each designated time point, the serum samples were carefully removed from the respective storage conditions and promptly stored at −80 °C to ensure long-term preservation and the maintenance of sample quality for subsequent experimental analyses.

#### 4.2.4. Effect of Freeze–Thaw Cycles on Glycosylated EV miRNAs in Serum

Fresh blood samples were collected from 3 healthy individuals using disposable serum collection tubes (BD, Franklin Lakes, NJ, USA). The serum was separated using the two-step centrifugation method, completed within a minimum of 30 min to a maximum of 90 min. One portion of the separated serum was directly used for glycosylated EV miRNA extraction, while the other portion was stored at −80 °C for 24 h before extracting the glycosylated EV miRNAs.

#### 4.2.5. Protection of Glycosylated EV miRNAs in Serum by Adding Protectants

Blood samples were collected from 3 healthy individuals using disposable serum collection tubes (BD, Franklin Lakes, NJ, USA). The serum was separated using the two-step centrifugation method, ensuring completion within a minimum of 30 min to a maximum of 90 min. Following separation, three groups of protectants were added to the serum: Protectant 1 (200 mM RVC), Protectant 2 (200 mM RVC supplemented with 1% PC300), and Protectant 3 (10 × normal saline). The protectants were diluted at a ratio of 1:10. The serum samples were then incubated at 25 °C for 0, 6, and 12 h. At each time point, the serum was removed and stored at −80 °C for subsequent experiments.

#### 4.2.6. Comparison of Glycosylated EV miRNAs in Plasma Separated by Three Different Collection Vessels

Fresh blood samples were obtained from healthy individuals using three different plasma collection tubes sourced from distinct manufacturers. The collection tubes included those containing EDTA anticoagulant (BD, Franklin Lakes, NJ, USA), sodium citrate (BD, Franklin Lakes, NJ, USA), and Lakebio cell-free RNA storage tubes (Lakebio, Hefei, China). Following blood collection, each type of collection tube was individually incubated at 25 °C for designated time intervals of 0, 4, 8, 16, 24, 48, 72, and 120 h. At each specified time point, two-step centrifugation was performed to separate the plasma, which was subsequently stored at −80 °C for subsequent experiments.

### 4.3. Glycosylated EV Isolation and Characterization

The Glyexo-Capture method was employed to specifically capture glycosylated EVs from serum/plasma and cell supernatant samples. This innovative Glyexo-Capture technology relies on the specific affinity between Lens culinaris lectin (LCA) and core fucose to facilitate the isolation of EVs from the collected blood samples, following a standardized protocol. The presence and characterization of EVs were investigated through comprehensive analyses, including TEM to visualize their morphological features, NTA to determine their size distribution, and WB analysis to detect specific EV-associated markers.

#### 4.3.1. TEM

Fixed in 2% glutaraldehyde at room temperature for 1 h, the EVs were subsequently stained with a uranyl acetate solution (pH 4.5). Visualization and examination of the EVs were performed using an FEI Tecnai Spirit transmission electron microscope (FEI, Eindhoven, The Netherlands) operating at 120 kV. Electron micrographs were captured employing a Gatan UltraScan 1000 charge-coupled device (CCD) camera (Pleasanton, CA, USA).

#### 4.3.2. NTA

The isolated EVs were appropriately diluted in phosphate-buffered saline (PBS) to achieve a specific concentration. NTA was conducted utilizing the Zeta View^®^ PMX120 instrument (Particle Metrix GmbH, Meerbusch, Germany) to evaluate the size distribution and quantity of the isolated particles. Subsequently, the acquired data were analyzed using dedicated NTA Build 127 software version 2.3 to analyze the movement characteristics of the particles.

#### 4.3.3. WB

Prior to conducting Western blot analysis, the EVs were subjected to lysis. The protein concentrations were determined using the Qubit Protein Assay Kit (Invitrogen, Pittsburgh, PA, USA) and the Qubit 4.0 Fluorometer (Invitrogen, Pittsburgh, PA, USA). A total of 10 µg of protein was loaded onto an SDS-polyacrylamide gel for electrophoresis. For the detection process, a rabbit anti-mouse IgG-HRP secondary antibody was utilized. The primary antibodies applied in this study included anti-CD81 (EXOAB-CD81A-1) (diluted 1:100, SBI, New York, NY, USA), anti-CD63 (EPR5702) (diluted 1:100, Abcam, Cambridge, MA, USA), anti-TSG101 (EPR7130(B)) (diluted 1:100, Abcam, Cambridge, MA, USA), and anti-calnexin (EPR3633(2)) (diluted 1:100, Abcam, Cambridge, MA, USA).

### 4.4. RNA Isolation and Analysis

#### 4.4.1. RNA Isolation

Total RNA was extracted from the isolated EVs utilizing the miRNeasy^®^ Mini Kit (Cat. 217,004, Qiagen, Hilden, Germany) following the recommended protocol provided by the manufacturer. Subsequently, the RNA concentration was determined using the Qubit microRNA Assay (Invitrogen, Pittsburgh, PA, USA) and the Qubit 4.0 Fluorometer (Invitrogen, Pittsburgh, PA, USA).

#### 4.4.2. RT-qPCR

A 10 μL mixture for the RT reaction was prepared, consisting of 2 μL 5× HiScript III Buffer, 0.5 μL HiScript III Reverse Transcriptase, 0.5 μL *E. coli* Poly(A) Polymerase, 1 μL ATP (10 mM), 1 μL Super pure dNTP (10 mM), 2 μL RT primer (10 μM), and 3 μL RNA. The RT reaction was conducted at 42 °C for 60 min, followed by a 5 min incubation at 85 °C.

qPCR was performed on an ABI 7500 Real-Time PCR System (Applied Biosystems, Foster City, CA, USA). The 25 μL PCR mixture comprised 12.5 μL 2× Robustart Premix Omni III (Probe qPCR), 2 μL PCR forward primer (10 μM), 2 μL PCR reverse primer (10 μM), 1 μL PCR probe (10 μM), 0.5 μL 50× Rox reference dye, 2 μL ddH_2_O, and 5 μL cDNA (5-fold diluted). The reaction commenced with a 5 min incubation at 95 °C, followed by 45 amplification cycles of 15 s at 95 °C and 32 s at 60 °C. The RT-qPCR primers used in this study were synthesized by Biotech (Shanghai, China) Co., Ltd. The sequences of primers and probes employed are presented in Table S1.

### 4.5. Statistical Analysis

The relative expression levels of miRNAs were determined using the 2^−ΔΔCt^ method. Statistical analysis was conducted using GraphPad Prism 9 software (GraphPad Software, San Diego, CA, USA). Data were analyzed for statistical significance using appropriate tests, such as Student’s *t*-test, one-way analysis of variance (ANOVA), or two-way ANOVA, as applicable. Results were considered significantly altered when *p* < 0.05.

## 5. Conclusions

In this study, we employed the GlyExo-Capture technique to isolate glycosylated EV miRNAs and assessed the impact of various sample processing methods, storage conditions, and environmental factors on the stability of glycosylated EV miRNAs. We recommend the two-step centrifugation method for experimental procedures. Serum should be promptly separated after blood collection, with separation required within 2 h at both 4 °C and 25 °C. Additionally, we evaluated the storage time of separated serum samples at 25 °C, 37 °C, 4 °C, and −20 °C. Higher temperatures corresponded to faster degradation of glycosylated EV miRNAs, necessitating shorter processing time, particularly during summer. Short-term storage is feasible at −20 °C for up to 3 months. Serum intended for glycosylated EV miRNA research should strictly avoid repeated freeze–thaw cycles and undergo only a single freeze–thaw cycle when stored at −80 °C. The preservatives we employed extend the time serum can be kept at room temperature, meeting the transportation and operational requirements of clinical experiments. The utilization of Lakebio’s cfRNA storage tubes better preserves the stability of miRNAs in glycosylated EVs in plasma. The stability of EV miRNAs safeguards the integrity of biomarkers, preventing their degradation and inactivation, thereby ensuring their reliable reflection of relevant biological processes or disease states in clinical research.

## Data Availability

Data generated and/or analyzed in this study are contained within the manuscript or the Supplementary Materials or can be requested from the corresponding authors.

## Supplementary Materials

The following supporting information can be downloaded at: https://www.mdpi.com/article/10.3390/molecules29010103/s1, Table S1: The sequence of primers and probes. Table S2: Degradation rate of four glycosylated EV miRNAs in three different serum separation methods at 8 h. Table S3: Degradation rate of four glycosylated EV miRNAs under different storage time after serum separation at 25 °C. Table S4: Degradation rate of four glycosylated EV miRNAs under different storage time after serum separation at 37 °C. Table S5: Degradation rate of four glycosylated EV miRNAs under different storage time after serum separation at 4 °C. Table S6: Relative expression levels of four glycosylated EV miRNAs at 12 h using three groups of protectants at 25 °C. Table S7: Statistical analysis information of four glycosylated EV miRNAs in three different serum separation methods. Table S8: Statistical analysis information of four glycosylated EV miRNAs under different storage time before serum separation. Table S9: Statistical analysis information of four glycosylated EV miRNAs under different storage time after serum separation. Table S10: Statistical analysis information of four glycosylated EV miRNAs after freeze-thaw. Table S11: Statistical analysis information of four glycosylated EV miRNAs using three groups of protectants at 25 °C. Table S12: Statistical analysis information of four glycosylated EV miRNAs using three different plasma collection vessels at 25 °C.
