# Supplementary material for: Storage Stability of Blood Samples for miRNAs in Glycosylated Extracellular Vesicles
Source: Molecules. 2023 Dec 23;29(1):103. doi: 10.3390/molecules29010103 (PMC10780163; doi:10.3390/molecules29010103)
Supplement: Supplementary file 1 [file molecules-29-00103-s001.zip › molecules-2765335-supplementary.pdf]

Table S1 The sequence of primers and probes.

Name	Sequence
RT primer	CGACTCGATCCAGTCTCAGGGTCCGAGGTATT<br>CGATCCTAACCCTCTCCTCGGTATCGAGTCGC<br>ACTTTTTTTTTTTTTTVN
Forward primer-let-7a	GCGCGTGAGGTAGTAGGTTGTATAGT
Forward primer-miR-16	GCCCGTAGCAGCACGTAAATATT
Forward primer-miR-125a	GCGCTCCCTGAGACCCTTT
Forward primer-miR-150	GCGCATCTCCCAACCCTT
Reverse primer	CAGTCTCAGGGTCCGAGGTATTC
Probe	TCGGTATCGAGTCGCACT

Table S2 Degradation rate of four glycosylated EV miRNAs in three different serum separation methods at 8h.

Treatments\miRNAs	let-7a	miR-16	miR-125a	miR-150
Treatment A	56%	36%	66%	77%
Treatment B	38%	5%	55%	58%
Treatment C	51%	13%	70%	65%

Treatment A: two-step centrifugation,Treatment B: rapid acceleration centrifugation, Treatment C: slow acceleration centrifugation,

Table S3 Degradation rate of four glycosylated EV miRNAs under different storage time after serum separation at 25℃.

Hours(h)\miRNAs	let-7a	miR-16	miR-125a	miR-150
0	0	0	0	0
4	24%	22%	38%	48%
8	50%	21%	42%	63%
12	58%	28%	48%	70%
24	78%	27%	82%	85%

Table S4 Degradation rate of four glycosylated EV miRNAs under different storage time after serum separation at 37℃.

Hours(h)\miRNAs	let-7a	miR-16	miR-125a	miR-150
0	0	0	0	0
2	50%	22%	29%	44%
4	75%	48%	63%	58%
6	83%	54%	76%	74%
8	92%	77%	89%	87%

10	92%	76%	89%	83%
12	91%	74%	88%	83%

Table S5 Degradation rate of four glycosylated EV miRNAs under different storage time after serum separation at 4℃.

Days(d)\miRNAs	let-7a	miR-16	miR-125a	miR-150
0	0	0	0	0
1	47%	38%	46%	43%
2	58%	38%	55%	55%
3	69%	55%	69%	69%
4	66%	63%	64%	61%
5	78%	78%	77%	78%
6	76%	80%	75%	75%

Table S6 Relative expression levels of four glycosylated EV miRNAs at 12h using three groups of protectants at 25℃.

miRNAs\groups	Protectant 1	Protectant 2	Protectant 3	Control group
let-7a	92%	98%	56%	47%
miR-16	89%	102%	56%	60%
miR-125a	109%	89%	47%	42%
miR-150	81%	98%	32%	27%
Average	93%	97%	47%	44%

Table S7 Statistical analysis information of four glycosylated EV miRNAs in three different serum separation methods.

miRNAs	Comparisons	Mean 1	Mean 2	Mean Diff.	95.00% CI of diff.	Below threshold?	Summary	Adjusted P Value
let-7a	Treatment A-0h vs. Treatment B-0h	1.082	0.923	0.1594	-0.1987 to 0.5175	No	ns	0.7951
	Treatment A-0h vs. Treatment C-0h	1.082	1.024	0.05793	-0.3219 to 0.4377	No	ns	0.9979
	Treatment B-0h vs. Treatment C-0h	0.923	1.024	-0.1014	-0.4812 to 0.2784	No	ns	0.9724
	Treatment A-8h vs. Treatment B-8h	0.4804	0.5718	-0.09143	-0.4593 to 0.2765	No	ns	0.9799
	Treatment A-8h vs. Treatment C-8h	0.4804	0.5056	-0.02528	-0.3834 to 0.3328	No	ns	>0.9999
	Treatment B-8h vs. Treatment C-8h	0.5718	0.5056	0.06614	-0.3018 to 0.4341	No	ns	0.9954
	Treatment A-0h vs. Treatment A-8h	1.082	0.4804	0.602	0.2439 to 0.9601	Yes	****	<0.0001
	Treatment B-0h vs. Treatment B-8h	0.923	0.5718	0.3512	-0.01674 to 0.7191	No	ns	0.0707
	Treatment C-0h vs. Treatment C-8h	1.024	0.5056	0.5187	0.1389 to 0.8986	Yes	**	0.0016
miR-16	Treatment A-0h vs. Treatment B-0h	1.071	0.8407	0.2307	-0.1274 to 0.5888	No	ns	0.4334
	Treatment A-0h vs. Treatment C-0h	1.071	1.048	0.02305	-0.3449 to 0.3910	No	ns	>0.9999
	Treatment B-0h vs. Treatment C-0h	0.8407	1.048	-0.2077	-0.5756 to 0.1602	No	ns	0.5832
	Treatment A-8h vs. Treatment B-8h	0.6904	0.8025	-0.112	-0.4800 to 0.2559	No	ns	0.9517
	Treatment A-8h vs. Treatment C-8h	0.6904	0.9089	-0.2184	-0.6075 to 0.1706	No	ns	0.5889
	Treatment B-8h vs. Treatment C-8h	0.8025	0.9089	-0.1064	-0.4862 to 0.2734	No	ns	0.9661
	Treatment A-0h vs. Treatment A-8h	1.071	0.6904	0.381	0.01307 to 0.7489	Yes	*	0.0376
	Treatment B-0h vs. Treatment B-8h	0.8407	0.8025	0.03822	-0.3199 to 0.3963	No	ns	0.9996
	Treatment C-0h vs. Treatment C-8h	1.048	0.9089	0.1395	-0.2496 to 0.5286	No	ns	0.9067
miR-125a	Treatment A-0h vs. Treatment B-0h	1.045	0.8533	0.1913	-0.1668 to 0.5493	No	ns	0.6406
	Treatment A-0h vs. Treatment C-0h	1.045	1.023	0.02139	-0.3921 to 0.4349	No	ns	>0.9999
	Treatment B-0h vs. Treatment C-0h	0.8533	1.023	-0.1699	-0.5834 to 0.2436	No	ns	0.8448

	Treatment A-8h vs. Treatment B-8h	0.3588	0.387	-0.02824	-0.3863 to 0.3299	No	ns	>0.9999
	Treatment A-8h vs. Treatment C-8h	0.3588	0.308	0.05075	-0.3073 to 0.4088	No	ns	0.9985
	Treatment B-8h vs. Treatment C-8h	0.387	0.308	0.07899	-0.2791 to 0.4371	No	ns	0.9882
	Treatment A-0h vs. Treatment A-8h	1.045	0.3588	0.6858	0.3277 to 1.044	Yes	****	<0.0001
	Treatment B-0h vs. Treatment B-8h	0.8533	0.387	0.4663	0.1082 to 0.8244	Yes	**	0.0032
	Treatment C-0h vs. Treatment C-8h	1.023	0.308	0.7152	0.3017 to 1.129	Yes	****	<0.0001
	Treatment A-0h vs. Treatment B-0h	1.095	0.822	0.2735	-0.08463 to 0.6316	No	ns	0.2433
	Treatment A-0h vs. Treatment C-0h	1.095	1.056	0.03992	-0.3736 to 0.4534	No	ns	0.9998
	Treatment B-0h vs. Treatment C-0h	0.822	1.056	-0.2336	-0.6470 to 0.1799	No	ns	0.5825
	Treatment A-8h vs. Treatment B-8h	0.2514	0.345	-0.09365	-0.5501 to 0.3628	No	ns	0.9916
miR-150	Treatment A-8h vs. Treatment C-8h	0.2514	0.3644	-0.113	-0.5021 to 0.2761	No	ns	0.9604
	Treatment B-8h vs. Treatment C-8h	0.345	0.3644	-0.01935	-0.4660 to 0.4273	No	ns	>0.9999
	Treatment A-0h vs. Treatment A-8h	1.095	0.2514	0.8441	0.4643 to 1.224	Yes	****	<0.0001
	Treatment B-0h vs. Treatment B-8h	0.822	0.345	0.477	0.03839 to 0.9155	Yes	*	0.0243
	Treatment C-0h vs. Treatment C-8h	1.056	0.3644	0.6912	0.2692 to 1.113	Yes	****	<0.0001

*P < 0.05, **P < 0.01, ****P < 0.0001, ns: nonsignificant.

Table S8 Statistical analysis information of four glycosylated EV miRNAs under different storage time before serum separation.

Temperature	miRNAs	Comparisons	Mean 1	Mean 2	Mean Diff.	95.00% CI of diff.	Below threshold?	Summary	Adjusted P Value
4℃	let-7a	0h vs. 4h	1.041	1.646	-0.6051	-1.560 to 0.3494	No	ns	0.3542
		0h vs. 8h	1.041	2.619	-1.578	-2.590 to -0.5653	Yes	***	0.0007

		0h vs. 12h	1.041	2.511	-1.47	-2.424 to -0.5153	Yes	***	0.0008
		0h vs. 24h	1.041	3.038	-1.997	-2.951 to -1.042	Yes	****	<0.0001
		0h vs. 48h	1.041	3.131	-2.089	-3.044 to -1.135	Yes	****	<0.0001
		0h vs. 4h	1.023	1.29	-0.2669	-1.220 to 0.6864	No	ns	0.9266
		0h vs. 8h	1.023	1.703	-0.6797	-1.633 to 0.2736	No	ns	0.2477
	miR-16	0h vs. 12h	1.023	1.512	-0.4887	-1.442 to 0.4646	No	ns	0.5547
		0h vs. 24h	1.023	1.811	-0.7875	-1.741 to 0.1658	No	ns	0.1397
		0h vs. 48h	1.023	1.65	-0.6267	-1.580 to 0.3266	No	ns	0.3186
		0h vs. 4h	1.019	0.8442	0.1746	-0.7787 to 1.128	No	ns	0.9871
		0h vs. 8h	1.019	0.8344	0.1844	-0.7689 to 1.138	No	ns	0.9837
	miR-125a	0h vs. 12h	1.019	0.7241	0.2947	-0.6586 to 1.248	No	ns	0.8946
		0h vs. 24h	1.019	0.5124	0.5065	-0.4468 to 1.460	No	ns	0.521
		0h vs. 48h	1.019	0.5046	0.5142	-0.4391 to 1.468	No	ns	0.5066
		0h vs. 4h	1.024	0.8233	0.2004	-0.7529 to 1.154	No	ns	0.9767
		0h vs. 8h	1.024	1.175	-0.1517	-1.105 to 0.8016	No	ns	0.9934
	miR-150	0h vs. 12h	1.024	1.169	-0.1453	-1.099 to 0.8080	No	ns	0.9944
		0h vs. 24h	1.024	1.428	-0.4045	-1.358 to 0.5488	No	ns	0.7165
		0h vs. 48h	1.024	1.511	-0.4875	-1.441 to 0.4658	No	ns	0.5569
		0h vs. 2h	1.04	1.159	-0.1193	-0.6836 to 0.4450	No	ns	0.9762
		0h vs. 4h	1.04	0.9111	0.1285	-0.4359 to 0.6928	No	ns	0.9675
25℃	let-7a	0h vs. 6h	1.04	0.8139	0.2257	-0.3387 to 0.7900	No	ns	0.7595
		0h vs. 8h	1.04	1.032	0.007954	-0.5564 to 0.5723	No	ns	>0.9999
		0h vs. 12h	1.04	0.8582	0.1813	-0.3830 to 0.7456	No	ns	0.8796

	miR-16	0h vs. 2h	1.021	1.311	-0.2903	-0.8546 to 0.2740	No	ns	0.5515
		0h vs. 4h	1.021	1.177	-0.1561	-0.7204 to 0.4082	No	ns	0.9299
		0h vs. 6h	1.021	1.207	-0.1861	-0.7504 to 0.3782	No	ns	0.8683
		0h vs. 8h	1.021	1.484	-0.4634	-1.028 to 0.1010	No	ns	0.1436
		0h vs. 12h	1.021	1.353	-0.3322	-0.8966 to 0.2321	No	ns	0.4226
	miR-125a	0h vs. 2h	1.021	1.22	-0.1997	-0.7640 to 0.3646	No	ns	0.8339
		0h vs. 4h	1.021	1.045	-0.02426	-0.5886 to 0.5401	No	ns	0.9999
		0h vs. 6h	1.021	0.899	0.1216	-0.4427 to 0.6860	No	ns	0.9741
		0h vs. 8h	1.021	0.8732	0.1474	-0.4169 to 0.7118	No	ns	0.9436
		0h vs. 12h	1.021	0.7116	0.309	-0.2553 to 0.8734	No	ns	0.4922
	miR-150	0h vs. 2h	1.009	0.8512	0.158	-0.4064 to 0.7223	No	ns	0.9267
		0h vs. 4h	1.009	0.9578	0.05136	-0.5130 to 0.6157	No	ns	0.9996
		0h vs. 6h	1.009	0.7622	0.247	-0.3174 to 0.8113	No	ns	0.6926
		0h vs. 8h	1.009	0.5957	0.4134	-0.1509 to 0.9777	No	ns	0.2258
		0h vs. 12h	1.009	0.7195	0.2897	-0.2747 to 0.8540	No	ns	0.5537

***P < 0.001, ****P < 0.0001, ns: nonsignificant.

Table S9 Statistical analysis information of four glycosylated EV miRNAs under different storage time after serum separation.

Temperature	miRNAs	Comparisons	Mean 1	Mean 2	Mean Diff.	95.00% CI of diff.	Below threshold?	Summary	Adjusted P Value
37℃	let-7a	0h vs. 2h	1.018	0.5068	0.5115	0.1511 to 0.8719	Yes	**	0.0022
		0h vs. 4h	1.018	0.2547	0.7636	0.4033 to 1.124	Yes	****	<0.0001

		0h vs. 6h	1.018	0.1697	0.8486	0.4883 to 1.209	Yes	****	<0.0001
		0h vs. 8h	1.018	0.07972	0.9386	0.5782 to 1.299	Yes	****	<0.0001
		0h vs. 10h	1.018	0.07864	0.9397	0.5793 to 1.300	Yes	****	<0.0001
		0h vs. 12h	1.018	0.08828	0.93	0.5697 to 1.290	Yes	****	<0.0001
	miR-16	0h vs. 2h	1.019	0.7946	0.2245	-0.1359 to 0.5849	No	ns	0.3789
		0h vs. 4h	1.019	0.5326	0.4865	0.1261 to 0.8468	Yes	**	0.004
		0h vs. 6h	1.019	0.4707	0.5484	0.1880 to 0.9088	Yes	***	0.001
		0h vs. 8h	1.019	0.2319	0.7872	0.4268 to 1.148	Yes	****	<0.0001
		0h vs. 10h	1.019	0.2464	0.7727	0.4123 to 1.133	Yes	****	<0.0001
		0h vs. 12h	1.019	0.2643	0.7548	0.3945 to 1.115	Yes	****	<0.0001
	miR-125a	0h vs. 2h	1.023	0.7218	0.3009	-0.05950 to 0.6612	No	ns	0.1346
		0h vs. 4h	1.023	0.3742	0.6484	0.2880 to 1.009	Yes	****	<0.0001
		0h vs. 6h	1.023	0.2419	0.7808	0.4204 to 1.141	Yes	****	<0.0001
		0h vs. 8h	1.023	0.1105	0.9121	0.5517 to 1.272	Yes	****	<0.0001
		0h vs. 10h	1.023	0.1126	0.91	0.5496 to 1.270	Yes	****	<0.0001
		0h vs. 12h	1.023	0.1235	0.8991	0.5387 to 1.259	Yes	****	<0.0001
	miR-150	0h vs. 2h	1.01	0.5668	0.4429	0.08255 to 0.8033	Yes	*	0.0101
		0h vs. 4h	1.01	0.4268	0.5829	0.2225 to 0.9433	Yes	***	0.0004
		0h vs. 6h	1.01	0.2603	0.7494	0.3890 to 1.110	Yes	****	<0.0001
		0h vs. 8h	1.01	0.1311	0.8786	0.5182 to 1.239	Yes	****	<0.0001
		0h vs. 10h	1.01	0.1697	0.84	0.4797 to 1.200	Yes	****	<0.0001
		0h vs. 12h	1.01	0.1695	0.8402	0.4798 to 1.201	Yes	****	<0.0001
25℃	let-7a	0h vs. 4h	1.026	0.7837	0.2422	-0.02211 to 0.5065	No	ns	0.0807

		0h vs. 8h	1.026	0.5095	0.5164	0.2521 to 0.7807	Yes	****	<0.0001
		0h vs. 12h	1.026	0.4355	0.5904	0.3260 to 0.8547	Yes	****	<0.0001
		0h vs. 24h	1.026	0.2225	0.8033	0.5390 to 1.068	Yes	****	<0.0001
	miR-16	0h vs. 4h	1.003	0.7839	0.2195	-0.04482 to 0.4838	No	ns	0.1278
		0h vs. 8h	1.003	0.7889	0.2144	-0.04986 to 0.4787	No	ns	0.1408
		0h vs. 12h	1.003	0.7243	0.2791	0.01476 to 0.5434	Yes	*	0.0357
		0h vs. 24h	1.003	0.7306	0.2728	0.008489 to 0.5371	Yes	*	0.0413
	miR-125a	0h vs. 4h	1.016	0.6312	0.3844	0.1201 to 0.6487	Yes	**	0.0024
		0h vs. 8h	1.016	0.5905	0.4251	0.1608 to 0.6894	Yes	***	0.0008
		0h vs. 12h	1.016	0.5267	0.4889	0.2246 to 0.7532	Yes	***	0.0001
		0h vs. 24h	1.016	0.1813	0.8344	0.5700 to 1.099	Yes	****	<0.0001
	miR-150	0h vs. 4h	1.004	0.5232	0.481	0.2167 to 0.7453	Yes	***	0.0002
		0h vs. 8h	1.004	0.3679	0.6362	0.3719 to 0.9005	Yes	****	<0.0001
		0h vs. 12h	1.004	0.3036	0.7005	0.4362 to 0.9648	Yes	****	<0.0001
		0h vs. 24h	1.004	0.1538	0.8504	0.5861 to 1.115	Yes	****	<0.0001
4℃	let-7a	0d vs. 1d	1.064	0.5636	0.5	0.1726 to 0.8274	Yes	***	0.0009
		0d vs. 2d	1.064	0.4511	0.6125	0.2851 to 0.9399	Yes	****	<0.0001
		0d vs. 3d	1.064	0.3269	0.7367	0.4093 to 1.064	Yes	****	<0.0001
		0d vs. 4d	1.064	0.3651	0.6984	0.3710 to 1.026	Yes	****	<0.0001
		0d vs. 5d	1.064	0.2385	0.8251	0.4977 to 1.152	Yes	****	<0.0001
		0d vs. 6d	1.064	0.2571	0.8065	0.4791 to 1.134	Yes	****	<0.0001
	miR-16	0d vs. 1d	1.016	0.6302	0.3857	0.05833 to 0.7131	Yes	*	0.0147
		0d vs. 2d	1.016	0.6281	0.3879	0.06046 to 0.7153	Yes	*	0.014

		0d vs. 3d	1.016	0.4581	0.5578	0.2304 to 0.8852	Yes	***	0.0002
		0d vs. 4d	1.016	0.3737	0.6422	0.3148 to 0.9696	Yes	****	<0.0001
		0d vs. 5d	1.016	0.2213	0.7946	0.4672 to 1.122	Yes	****	<0.0001
		0d vs. 6d	1.016	0.2066	0.8093	0.4819 to 1.137	Yes	****	<0.0001
	miR-125a	0d vs. 1d	1.074	0.5822	0.4915	0.1641 to 0.8189	Yes	**	0.0012
		0d vs. 2d	1.074	0.4788	0.5949	0.2675 to 0.9223	Yes	****	<0.0001
		0d vs. 3d	1.074	0.334	0.7398	0.4124 to 1.067	Yes	****	<0.0001
		0d vs. 4d	1.074	0.384	0.6897	0.3623 to 1.017	Yes	****	<0.0001
		0d vs. 5d	1.074	0.2486	0.8251	0.4977 to 1.153	Yes	****	<0.0001
		0d vs. 6d	1.074	0.2737	0.8001	0.4727 to 1.127	Yes	****	<0.0001
	miR-150	0d vs. 1d	1.058	0.6004	0.4578	0.1304 to 0.7852	Yes	**	0.0027
		0d vs. 2d	1.058	0.4756	0.5826	0.2552 to 0.9100	Yes	***	0.0001
		0d vs. 3d	1.058	0.3238	0.7344	0.4070 to 1.062	Yes	****	<0.0001
		0d vs. 4d	1.058	0.4086	0.6496	0.3222 to 0.9770	Yes	****	<0.0001
		0d vs. 5d	1.058	0.2321	0.8261	0.4987 to 1.154	Yes	****	<0.0001
		0d vs. 6d	1.058	0.2612	0.797	0.4696 to 1.124	Yes	****	<0.0001
-20℃	let-7a	0d vs. 10d	1.009	1.079	-0.06976	-0.4648 to 0.3253	No	ns	0.9974
		0d vs. 20d	1.009	1.044	-0.03491	-0.4300 to 0.3602	No	ns	0.9997
		0d vs. 30d	1.009	0.9954	0.01398	-0.3811 to 0.4091	No	ns	0.9999
		0d vs. 40d	1.009	1.067	-0.05762	-0.4527 to 0.3375	No	ns	0.9994
		0d vs. 50d	1.009	0.9868	0.02256	-0.3725 to 0.4176	No	ns	0.9998
		0d vs. 60d	1.009	1.052	-0.04312	-0.4382 to 0.3520	No	ns	0.9996
		0d vs. 70d	1.009	1.031	-0.02209	-0.4172 to 0.3730	No	ns	0.9998

	0d vs. 80d	1.009	1.154	-0.1447	-0.5398 to 0.2504	No	ns	0.9032
	0d vs. 90d	1.009	1.081	-0.07197	-0.4670 to 0.3231	No	ns	0.9972
	0d vs. 10d	1.02	1.081	-0.06083	-0.4559 to 0.3343	No	ns	0.9994
	0d vs. 20d	1.02	1.132	-0.1113	-0.5064 to 0.2838	No	ns	0.9772
	0d vs. 30d	1.02	1.148	-0.1272	-0.5223 to 0.2679	No	ns	0.9503
	0d vs. 40d	1.02	1.07	-0.0493	-0.4444 to 0.3458	No	ns	0.9995
miR-16	0d vs. 50d	1.02	0.9764	0.04405	-0.3510 to 0.4391	No	ns	0.9996
	0d vs. 60d	1.02	0.988	0.03245	-0.3626 to 0.4275	No	ns	0.9997
	0d vs. 70d	1.02	1.118	-0.09746	-0.4925 to 0.2976	No	ns	0.9904
	0d vs. 80d	1.02	1.063	-0.04237	-0.4375 to 0.3527	No	ns	0.9996
	0d vs. 90d	1.02	0.8794	0.141	-0.2541 to 0.5361	No	ns	0.9147
	0d vs. 10d	1.01	1.076	-0.0661	-0.4612 to 0.3290	No	ns	0.9993
	0d vs. 20d	1.01	1.103	-0.09353	-0.4886 to 0.3016	No	ns	0.9921
	0d vs. 30d	1.01	0.9474	0.06216	-0.3329 to 0.4572	No	ns	0.9994
	0d vs. 40d	1.01	0.9989	0.01075	-0.3843 to 0.4058	No	ns	>0.9999
miR-125a	0d vs. 50d	1.01	0.9302	0.07939	-0.3157 to 0.4745	No	ns	0.9967
	0d vs. 60d	1.01	1.027	-0.01752	-0.4126 to 0.3776	No	ns	0.9999
	0d vs. 70d	1.01	0.9267	0.0829	-0.3122 to 0.4780	No	ns	0.9964
	0d vs. 80d	1.01	1.115	-0.1055	-0.5006 to 0.2895	No	ns	0.9841
	0d vs. 90d	1.01	1.062	-0.05223	-0.4473 to 0.3429	No	ns	0.9995
	0d vs. 10d	1.014	1.082	-0.06865	-0.4637 to 0.3264	No	ns	0.9976
miR-150	0d vs. 20d	1.014	1.023	-0.009831	-0.4049 to 0.3853	No	ns	>0.9999
	0d vs. 30d	1.014	1.021	-0.007688	-0.4028 to 0.3874	No	ns	>0.9999

	0d vs. 40d	1.014	1.04	-0.02661	-0.4217 to 0.3685	No	ns	0.9997
	0d vs. 50d	1.014	1.005	0.008576	-0.3865 to 0.4037	No	ns	>0.9999
	0d vs. 60d	1.014	1.016	-0.002271	-0.3974 to 0.3928	No	ns	>0.9999
	0d vs. 70d	1.014	1.011	0.002163	-0.3929 to 0.3972	No	ns	>0.9999
	0d vs. 80d	1.014	1.019	-0.005085	-0.4002 to 0.3900	No	ns	>0.9999
	0d vs. 90d	1.014	1.074	-0.06008	-0.4552 to 0.3350	No	ns	0.9994

*P < 0.05, **P < 0.01, ***P < 0.001, ****P < 0.0001, ns: nonsignificant.

Table S10 Statistical analysis information of four glycosylated EV miRNAs after freeze-thaw.

miRNAs	Mean 1	Mean 2	Mean Diff.	95.00% CI of diff.	Below threshold?	Summary	Adjusted P Value
let-7a	1.025	0.308	0.7168	0.2300 to 1.204	Yes	**	0.0031
miR-16	1.016	0.5257	0.4902	0.003371 to 0.9771	Yes	*	0.0481
miR-125a	1.044	0.3233	0.7205	0.2336 to 1.207	Yes	**	0.003
miR-150	1.026	0.3028	0.7236	0.2367 to 1.210	Yes	**	0.0029

*P < 0.05, **P < 0.01.

Table S11 Statistical analysis information of four glycosylated EV miRNAs using three groups of protectants at 25℃.

miRNAs	Groups	Comparisons	Mean 1	Mean 2	Mean Diff.	95.00% CI of diff.	Below threshold?	Summary	Adjusted P Value
let-7a	Protectant 1	0h vs. 6h	1.014	0.8583	0.1556	-0.1736 to 0.4849	No	ns	0.444
		0h vs. 12h	1.014	0.9304	0.08345	-0.2458 to 0.4127	No	ns	0.7779

	Protectant 2	0h vs. 6h	1.008	0.8528	0.1551	-0.1741 to 0.4843	No	ns	0.4462
		0h vs. 12h	1.008	0.9845	0.02338	-0.3059 to 0.3526	No	ns	0.9798
	Protectant 3	0h vs. 6h	1.025	0.4859	0.5395	0.2103 to 0.8688	Yes	**	0.0015
		0h vs. 12h	1.025	0.57	0.4554	0.1261 to 0.7846	Yes	**	0.0065
miR-16	Control group	0h vs. 6h	1.015	0.645	0.3697	0.04045 to 0.6989	Yes	*	0.0267
		0h vs. 12h	1.015	0.4818	0.5329	0.2036 to 0.8621	Yes	**	0.0017
	Protectant 1	0h vs. 6h	1.017	0.9907	0.02674	-0.3385 to 0.3919	No	ns	0.9786
		0h vs. 12h	1.017	0.9081	0.1093	-0.2559 to 0.4745	No	ns	0.7077
	Protectant 2	0h vs. 6h	1.022	0.9438	0.07813	-0.2871 to 0.4433	No	ns	0.8346
		0h vs. 12h	1.022	1.047	-0.02466	-0.3899 to 0.3405	No	ns	0.9817
	Protectant 3	0h vs. 6h	1.008	0.6085	0.3998	0.03464 to 0.7651	Yes	*	0.0309
		0h vs. 12h	1.008	0.5603	0.4481	0.08285 to 0.8133	Yes	*	0.0154
miR-125a	Control group	0h vs. 6h	1.017	0.7532	0.2638	-0.1014 to 0.6290	No	ns	0.1779
		0h vs. 12h	1.017	0.6134	0.4037	0.03848 to 0.7689	Yes	*	0.0293
	Protectant 1	0h vs. 6h	1.017	1.04	-0.02279	-0.3312 to 0.2856	No	ns	0.9781
		0h vs. 12h	1.017	1.104	-0.08627	-0.3946 to 0.2221	No	ns	0.7384
	Protectant 2	0h vs. 6h	1.01	0.9586	0.05184	-0.2565 to 0.3602	No	ns	0.8934
		0h vs. 12h	1.01	0.9035	0.1069	-0.2014 to 0.4153	No	ns	0.6333
	Protectant 3	0h vs. 6h	1.023	0.7421	0.2812	-0.02716 to 0.5896	No	ns	0.0766
		0h vs. 12h	1.023	0.4768	0.5466	0.2382 to 0.8549	Yes	***	0.0007
	Control group	0h vs. 6h	1.014	0.8268	0.1868	-0.1215 to 0.4952	No	ns	0.2812
		0h vs. 12h	1.014	0.4278	0.5858	0.2775 to 0.8942	Yes	***	0.0003
miR-150	Protectant 1	0h vs. 6h	1.01	0.9195	0.09005	-0.1953 to 0.3754	No	ns	0.6825

	0h vs. 12h	1.01	0.8158	0.1937	-0.09166 to 0.4791	No	ns	0.2123
Protectant 2	0h vs. 6h	1.01	1.005	0.0052	-0.2802 to 0.2906	No	ns	0.9986
	0h vs. 12h	1.01	0.9893	0.02054	-0.2648 to 0.3059	No	ns	0.9792
Protectant 3	0h vs. 6h	1.012	0.3981	0.6143	0.3289 to 0.8997	Yes	****	<0.0001
	0h vs. 12h	1.012	0.3215	0.6908	0.4054 to 0.9762	Yes	****	<0.0001
Control group	0h vs. 6h	1.008	0.4506	0.5575	0.2721 to 0.8429	Yes	***	0.0002
	0h vs. 12h	1.008	0.2759	0.7321	0.4467 to 1.017	Yes	****	<0.0001

*P < 0.05, **P < 0.01, ***P < 0.001, ****P < 0.0001, ns: nonsignificant.

Table S12 Statistical analysis information of four glycosylated EV miRNAs using three different plasma collection vessels at 25℃.

Groups	miRNAs	Comparisons	Mean 1	Mean 2	Mean Diff.	95.00% CI of diff.	Below threshold?	Summary	Adjusted P Value
Sodium citrate	let-7a	0h vs. 4h	1.057	1.754	-0.6975	-2.822 to 1.428	No	ns	0.9106
		0h vs. 8h	1.057	2.021	-0.9638	-3.089 to 1.161	No	ns	0.7075
		0h vs. 16h	1.057	2.024	-0.9672	-3.092 to 1.158	No	ns	0.7045
		0h vs. 24h	1.057	1.925	-0.8682	-2.993 to 1.257	No	ns	0.7907
		0h vs. 48h	1.057	1.991	-0.9335	-3.059 to 1.191	No	ns	0.7346
		0h vs. 72h	1.057	4.278	-3.221	-5.346 to -1.096	Yes	**	0.001
		0h vs. 120h	1.057	12.57	-11.51	-13.64 to -9.387	Yes	****	<0.0001
	miR-16	0h vs. 4h	1.027	1.261	-0.2338	-3.612 to 3.145	No	ns	0.9997
		0h vs. 8h	1.027	1.277	-0.2505	-3.629 to 3.128	No	ns	0.9997
		0h vs. 16h	1.027	1.535	-0.5078	-3.886 to 2.871	No	ns	0.9978

		0h vs. 24h	1.027	1.769	-0.7421	-4.121 to 2.637	No	ns	0.9884
		0h vs. 48h	1.027	2.204	-1.177	-4.556 to 2.201	No	ns	0.8848
		0h vs. 72h	1.027	5.257	-4.23	-7.608 to -0.8511	Yes	**	0.0084
		0h vs. 120h	1.027	9.607	-8.58	-11.96 to -5.201	Yes	****	<0.0001
		0h vs. 4h	1.047	0.8626	0.1846	-2.445 to 2.815	No	ns	0.9997
		0h vs. 8h	1.047	0.8768	0.1703	-2.460 to 2.800	No	ns	0.9997
		0h vs. 16h	1.047	0.8399	0.2072	-2.423 to 2.837	No	ns	0.9997
	miR-125a	0h vs. 24h	1.047	0.7628	0.2843	-2.346 to 2.914	No	ns	0.9996
		0h vs. 48h	1.047	1.045	0.002269	-2.628 to 2.632	No	ns	>0.9999
		0h vs. 72h	1.047	1.228	-0.1807	-2.811 to 2.449	No	ns	0.9997
		0h vs. 120h	1.047	2.582	-1.535	-4.165 to 1.095	No	ns	0.4578
		0h vs. 4h	1.057	0.8778	0.1788	-2.212 to 2.570	No	ns	0.9997
		0h vs. 8h	1.057	0.8819	0.1747	-2.217 to 2.566	No	ns	0.9997
		0h vs. 16h	1.057	0.8141	0.2425	-2.149 to 2.634	No	ns	0.9996
	miR-150	0h vs. 24h	1.057	0.7406	0.316	-2.075 to 2.707	No	ns	0.9994
		0h vs. 48h	1.057	1.081	-0.02407	-2.415 to 2.367	No	ns	>0.9999
		0h vs. 72h	1.057	1.155	-0.09794	-2.489 to 2.293	No	ns	0.9999
		0h vs. 120h	1.057	2.594	-1.537	-3.928 to 0.8542	No	ns	0.3583
		0h vs. 4h	1.046	1.036	0.01033	-2.115 to 2.135	No	ns	>0.9999
		0h vs. 8h	1.046	0.8907	0.1557	-1.969 to 2.281	No	ns	0.9997
EDTA	let-7a	0h vs. 16h	1.046	1.581	-0.5342	-2.659 to 1.591	No	ns	0.975
		0h vs. 24h	1.046	1.557	-0.5105	-2.635 to 1.615	No	ns	0.9803
		0h vs. 48h	1.046	1.132	-0.08593	-2.211 to 2.039	No	ns	0.9999

	0h vs. 72h	1.046	1.523	-0.477	-2.602 to 1.648	No	ns	0.9867
	0h vs. 120h	1.046	1.633	-0.5868	-2.712 to 1.538	No	ns	0.9598
	0h vs. 4h	1.06	1.094	-0.03415	-3.413 to 3.345	No	ns	>0.9999
	0h vs. 8h	1.06	1.188	-0.1278	-3.506 to 3.251	No	ns	0.9999
	0h vs. 16h	1.06	2.918	-1.858	-5.237 to 1.521	No	ns	0.5203
miR-16	0h vs. 24h	1.06	3.705	-2.645	-6.024 to 0.7333	No	ns	0.1815
	0h vs. 48h	1.06	2.484	-1.424	-4.802 to 1.955	No	ns	0.7678
	0h vs. 72h	1.06	3.348	-2.288	-5.667 to 1.091	No	ns	0.3069
	0h vs. 120h	1.06	1.28	-0.2196	-3.598 to 3.159	No	ns	0.9997
	0h vs. 4h	1.008	0.5845	0.4232	-2.207 to 3.053	No	ns	0.9975
	0h vs. 8h	1.008	0.7972	0.2105	-2.419 to 2.841	No	ns	0.9997
	0h vs. 16h	1.008	0.8454	0.1623	-2.468 to 2.792	No	ns	0.9997
miR-125a	0h vs. 24h	1.008	1.104	-0.09591	-2.726 to 2.534	No	ns	0.9999
	0h vs. 48h	1.008	11.83	-10.82	-13.45 to -8.194	Yes	****	<0.0001
	0h vs. 72h	1.008	9.667	-8.659	-11.29 to -6.029	Yes	****	<0.0001
	0h vs. 120h	1.008	6.076	-5.068	-7.698 to -2.438	Yes	****	<0.0001
	0h vs. 4h	1.013	0.5855	0.4276	-1.964 to 2.819	No	ns	0.9953
	0h vs. 8h	1.013	0.7818	0.2312	-2.160 to 2.622	No	ns	0.9996
	0h vs. 16h	1.013	0.8976	0.1155	-2.276 to 2.507	No	ns	0.9998
miR-150	0h vs. 24h	1.013	1.051	-0.03789	-2.429 to 2.353	No	ns	>0.9999
	0h vs. 48h	1.013	10.42	-9.406	-11.80 to -7.015	Yes	****	<0.0001
	0h vs. 72h	1.013	10.34	-9.323	-11.71 to -6.932	Yes	****	<0.0001
	0h vs. 120h	1.013	6.347	-5.334	-7.725 to -2.942	Yes	****	<0.0001

Lakebio	let-7a	0h vs. 4h	1.099	0.9258	0.1732	-1.952 to 2.298	No	ns	0.9997
		0h vs. 8h	1.099	0.8292	0.2698	-1.855 to 2.395	No	ns	0.9995
		0h vs. 16h	1.099	0.8795	0.2195	-1.905 to 2.345	No	ns	0.9996
		0h vs. 24h	1.099	0.7345	0.3645	-1.761 to 2.489	No	ns	0.9971
		0h vs. 48h	1.099	1.483	-0.3835	-2.509 to 1.741	No	ns	0.9951
		0h vs. 72h	1.099	1.031	0.06832	-2.057 to 2.193	No	ns	0.9999
		0h vs. 120h	1.099	1.313	-0.2142	-2.339 to 1.911	No	ns	0.9996
	miR-16	0h vs. 4h	1.018	0.765	0.2529	-3.126 to 3.632	No	ns	0.9997
		0h vs. 8h	1.018	0.8629	0.155	-3.224 to 3.534	No	ns	0.9998
		0h vs. 16h	1.018	0.8178	0.2001	-3.179 to 3.579	No	ns	0.9998
		0h vs. 24h	1.018	0.7974	0.2205	-3.158 to 3.599	No	ns	0.9997
		0h vs. 48h	1.018	2.837	-1.819	-5.198 to 1.559	No	ns	0.542
		0h vs. 72h	1.018	2.709	-1.691	-5.070 to 1.688	No	ns	0.6159
		0h vs. 120h	1.018	2.04	-1.022	-4.401 to 2.357	No	ns	0.9378
	miR-125a	0h vs. 4h	1.021	0.6966	0.3241	-2.306 to 2.954	No	ns	0.9995
		0h vs. 8h	1.021	0.787	0.2337	-2.396 to 2.864	No	ns	0.9997
		0h vs. 16h	1.021	0.7053	0.3153	-2.315 to 2.945	No	ns	0.9995
		0h vs. 24h	1.021	0.8841	0.1366	-2.493 to 2.767	No	ns	0.9998
		0h vs. 48h	1.021	0.8581	0.1626	-2.467 to 2.793	No	ns	0.9997
		0h vs. 72h	1.021	0.8653	0.1554	-2.475 to 2.785	No	ns	0.9998
		0h vs. 120h	1.021	0.9451	0.07559	-2.554 to 2.706	No	ns	0.9999
	miR-150	0h vs. 4h	1.021	0.7578	0.2634	-2.128 to 2.655	No	ns	0.9996
		0h vs. 8h	1.021	0.8685	0.1527	-2.239 to 2.544	No	ns	0.9997

	0h vs. 16h	1.021	0.7034	0.3178	-2.073 to 2.709	No	ns	0.9994
	0h vs. 24h	1.021	0.9013	0.1199	-2.271 to 2.511	No	ns	0.9998
	0h vs. 48h	1.021	0.8496	0.1717	-2.220 to 2.563	No	ns	0.9997
	0h vs. 72h	1.021	0.8893	0.1319	-2.259 to 2.523	No	ns	0.9998
	0h vs. 120h	1.021	0.9961	0.0251	-2.366 to 2.416	No	ns	>0.9999

**P < 0.01, ****P < 0.0001, ns: nonsignificant.
